# Prevalence of undetected chronic kidney disease in high-risk middle-aged patients in primary care: a cross-sectional study

**DOI:** 10.3389/fmed.2024.1412689

**Published:** 2024-08-13

**Authors:** Andrea Siebenhofer, Christine Loder, Alexander Avian, Elisabeth Platzer, Carolin Zipp, Astrid Mauric, Ulrike Spary-Kainz, Andrea Berghold, Alexander R. Rosenkranz

**Affiliations:** ^1^Institute of General Practice and Evidence-based Health Services Research, Medical University of Graz, Graz, Austria; ^2^Institute for General Practice, Goethe University Frankfurt am Main, Frankfurt, Germany; ^3^Institute for Medical Informatics, Statistics and Documentation, Medical University of Graz, Graz, Austria; ^4^Clinical Division of Nephrology, Department of Internal Medicine, Medical University of Graz, Graz, Austria

**Keywords:** prevalence, chronic kidney disease, middle-aged, primary care, cross-sectional study

## Abstract

**Introduction:**

The global health burden of chronic kidney disease (CKD) results from both the disease itself and the numerous health problems associated with it. The aim of this study was to estimate the prevalence of previously undetected CKD in middle-aged patients with risk factors for CKD. Identified patients were included in the Styrian nephrology awareness program “kidney.care 2.0” and data on their demographics, risk factors and kidney function were described.

**Methods:**

Cross-sectional analysis of baseline data derived from the “kidney.care 2.0” study of 40–65 year old patients with at least one risk factor for CKD (hypertension, diabetes, cardiovascular disease, obesity or family history of end-stage kidney disease). Participants were considered to have previously undetected CKD if their estimated glomular filtration rate (eGFR) was less than 60 ml/min/1.73 m^2^ and/or albumin creatinine ratio (ACR) ≥ 30 mg/g. We calculated the prevalence of previously undetected CKD and performed multivariate analyses.

**Results:**

A total of 749 participants were included in this analysis. The prevalence of previously undetected CKD in an at-risk population was estimated at 20.1% (95%CI: 17.1–23.6). Multivariable analysis showed age (OR 1.06, 95%CI: 1.02–1.09), diabetes mellitus (OR 1.65, 95%CI: 1.12–2.30) and obesity (OR: 1.55, 95%CI: 1.04–2.30) to be independent predictors of CKD. The majority of patients with previously undetected CKD had category A2-A3 albuminuria (121 out of 150). Most patients with previously undetected eGFR < 60 ml/min/1.73 m^2^ were in stage G3 (36 out of 39 patients).

**Discussion:**

Pragmatic, targeted, risk-based screening for CKD in primary care successfully identified a significant number of middle-aged patients with previously undetected CKD and addressed the problem of these patients being overlooked for future optimized care. The intervention may slow progression to kidney failure and prevent related cardiovascular events.

## Introduction

The global health burden of chronic kidney disease (CKD) results from both the disease itself and the numerous health problems including cardiovascular issues that are associated with it (1–3). Almost one billion people worldwide (4) suffer from kidney disease, with prevalence across the world varying from around 3 to 18%, depending on country (5, 6). Many risk factors such as diabetes, hypertension, and obesity (7, 8) are associated with increased prevalence of CKD (9, 10) as is multimorbidity (9, 11).

Not only is early-stage CKD often asymptomatic (12), but despite laboratory evidence it frequently remains undiagnosed (13–15). In a large study of the digital health records of millions of patients, two-thirds of patients whose laboratory data indicated the presence of CKD had not been diagnosed as having the disease (13). Based on digital records from five countries, Tangri et al. demonstrated that most cases of stage G3 CKD (estimated glomular filtration rate (eGFR) ≥ 30 and < 60 ml/min/1.73 m^2^) are not diagnosed and lack an International Classification of Diseases (ICD) 9/10 diagnosis code, despite the documentation of reduced GFR in patients’ records (14). Clinical data from a huge population-based cohort conducted as part of the Study of Health in Pomerania (SHIP-START) showed that only 5% of patients had an ICD code for a diagnosis of CKD although 20% had an eGFR < 60 ml/min/1.73 m^2^ 60 or albuminuria ≥ 30 mg/gl (15). In a systematic review from 2020, Neale et al. (16) explain that a lack of ICD coding does not necessarily mean CDK is not noticed and provide a number of reasons for it. These include CKD not being the main medical problem, combined with a lack of time, limited access to specialist nephrologists, software systems that do not automatically flag abnormal results, fear of frightening patients by diagnosing such an illness, and concerns about stigmatization. Last but not least, diagnostic challenges may further result from unclear definitions of CKD and consequent dissatisfaction with CKD guidelines (16). An analysis by Friedl et al. (17), which compared routine laboratory parameters with the actual documentation of ICD-10 diagnoses of CKD in patients’ discharge reports, also showed that CKD often goes undetected, even in a hospital setting (17). Another study that evaluated over 9,000 patients in a primary care setting showed that > 50 % of CKD stage G3-5 patients were not diagnosed with CKD and received a lower level of care than patients that had been diagnosed with CKD (18). Most people with mild CKD presented in primary care practices, and in several countries initiatives aimed at improving the identification and management of CKD already exist (19–22).

Beneficial screening programs should do more good than harm (23). For CKD, many bodies such as NICE (24), the American College of Physicians (ACP) (25), the US Preventive Service Task Force (USPSTF) (26) and the Agency of Healthcare Research and Quality (27) have recommended against population-wide screening due to concerns about overdiagnosis, unnecessary treatment of normal age-related decline in kidney function, a high number of tests associated with further event-related diagnostic investigations, and excessive cost. In 2021, the conclusions of the Kidney Disease: Improving Global Outcomes (KDIGO) controversies conference were published, which recommended CKD screening coupled with risk stratification in a primary or community care setting, followed by immediate treatment for high-risk individuals (28). The procedure of filtering out high-risk patients prior to conducting laboratory tests has been evaluated in several studies over the past 20 years (19, 21, 29). It has also recently been carried out in the Pan-Canadian See Kidney Disease (SeeKD) Targeted Screening Program, in which patients with at least one risk factor underwent laboratory tests and were provided with subsequent information and tailored treatment (20).

In Austria, the nephrology awareness program “niere.schützen” (“kidney.care”) was launched in a primary care setting in the state of Styria for people at risk of kidney failure (30). General practitioners (GPs) were routinely provided with evidence-based information materials on diagnosis, treatment and referral procedures, but only a minority of GPs participated in the awareness program. The opinions of Styrian GPs were evaluated and several factors aimed at increasing its attractiveness were identified. These included further education for doctors, the need for more contacts among nephrologists, and financial incentives for carrying out laboratory tests (31). At the same time, we searched for existing international nephrological screening and support programs in order to identify further evaluation parameters and screening concepts (32). Finally, in January 2021, we launched “niere.schützen 2.0” (“kidney.care 2.0”), with the aim of not only raising awareness among GPs, but enhancing communication between GPs and nephrologists, improving patient education, and estimating the prevalence of CKD in a risk population (33). In line with recommendations on program evaluation, we conducted patient-centered labeling (34).

The primary objective of the study described in this manuscript was to estimate the prevalence of previously undetected CKD in middle-aged patients with risk factors for CKD (Table 1). Our secondary aim was to classify participants in the Styrian nephrology awareness program “kidney.care 2.0” in terms of their demographics, risk factors and kidney function.

**TABLE 1:** Risk factors for CKD according to the “kidney.care” program.

Arterial hypertension (documented diagnosis of hypertension for at least 3 months, defined as systolic blood pressure > 140 or diastolic blood pressure > 90 mmHg)
Diabetes mellitus (defined as type 1 and type 2)
Obesity (defined as body mass index > 30 kg/m^2^)
History of cardiovascular diseases (stroke, transient ischemic attack (TIA), coronary heart disease, peripheral arterial disease)
End-stage kidney disease in the family (family defined as children, parents and siblings)

## 2 Materials and methods

### 2.1 Study design and outcomes

We conducted a cross-sectional analysis of baseline data from the “kidney.care 2.0” study. Over a 24-month period lasting from January 2021 to December 2022, GPs in Styria (Austria) screened patients aged 40–65 years for the presence of at least one CKD risk factor (Table 1). Each GP was expected to screen all at-risk patients attending his or her practice over an 8-week period, which could be extended if necessary in consultation with the study team, as was frequently the case due to the COVID pandemic in 2020–2022 (34).

Inclusion criteria were men and women of all ethnic groups, aged between 40 and 65 years, treated by a GP with a health insurance contract, the provision of written consent to participate in the study, and with at least one of the risk factors for CKD (Table 1).

Patients were excluded if GPs’ records showed they had a prior diagnosis of CKD, they had previously participated in the “kidney.care” program, had an eGFR lower than 15 ml/min/1.73 m^2^, had received a kidney transplant, dialysis or cancer treatment, or had New York Heart Association (NYHA) heart failure > stage II. Patients with a life expectancy of less than six months or who were unable to provide informed consent according to their GP were also excluded.

For this study, participants were diagnosed with CKD if the laboratory results indicated a decrease in kidney function (eGFR < 60 ml/min/1.73 m^2^) and/or evidence of kidney damage (albumin creatinine ratio (ACR) ≥ 30 mg/g). In the case of initial kidney damage (ACR ≥ 30 mg/g), a second ACR was assessed within three months and CKD was diagnosed only if an albuminuria test was positive.

The primary outcome, based on a descriptive analysis of the baseline data of the “kidney.care 2.0” study, was the prevalence of previously undetected CKD in middle-aged at-risk patients in primary care practices. The participants’ demographic characteristics, risk factors, laboratory results and concomitant medication served as secondary outcomes.

The study protocol was approved by the Ethics Committee of the Medical University of Graz (reference 32-554 ex 19/20) and registered with the German Clinical Trials Register (registration number DRKS00022966). The patients/participants provided their written informed consent to participate in this study.

### 2.2 Recruitment and screening

To achieve the required number of 30 to 40 collaborating GPs, a variety of recruitment strategies were developed and implemented between January 2021 and December 2022. Participating GPs were asked to consecutively screen patients aged 40–65 with the defined risk factors that presented to the practice during an eight-week period (to avoid selection bias) and satisfied all other eligibility criteria. Recruitment at each individual practice ceased when 24–40 eligible patients had agreed and provided written informed consent to participate in the “kidney.care 2.0 study”. If necessary, it was possible to extend the eight-week period in consultation with the study team, provided that it ended no later than December 31, 2022. For further details see Supplementary Table 1.

### 2.3 Data collection and patient management

Eligible patients that had provided their written informed consent attended an initial baseline visit at which the study was explained to them. At the baseline visit, blood and urine samples were collected, sent for laboratory testing and analyzed in seven laboratories that used isotope dilution mass spectrometry (IDMS) to measure serum creatinine (35). The eGFR was then reported using the CKD Epidemiology Collaboration (CKD-EPI) equation (36), and ACR was assessed. In accordance with guidelines, the eGFR values were rounded to the nearest whole number for categorization (37).

**FIGURE 1:**
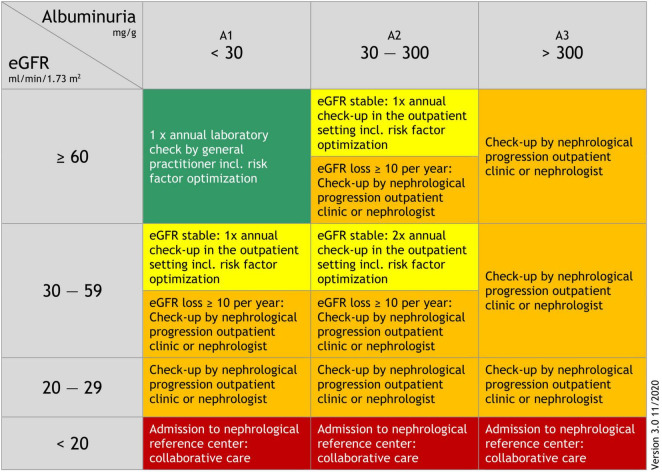
Kidney-care referral schema.

Based on the Kidney Failure Risk Equation (38), we developed an adapted and slightly simplified kidney-care referral schema (Figure 1) which was piloted with GPs and nephrologists from the Division of Nephrology, Medical University of Graz for practicability and comprehensibility (30). Prior to participation in our “kidney.care 2.0” program all GPs participated in a short training course and were provided with guidance materials for treatment and further management in accordance with the Clinical Practice Guideline for the Evaluation and Management of Chronic Kidney Disease (KDIGO) (37). In addition, we set up a “progression outpatient clinic” at the Division of Nephrology, Medical University of Graz, to ensure that patients could be referred there if necessary. Participating GPs also had the opportunity to discuss treatment options with a nephrologist via a dedicated “telephone hotline”.

After 12 months, all patients, irrespective of whether they had been diagnosed with CKD or not, were invited by their GP to a follow-up consultation at their primary care practice to evaluate changes in kidney function and the need for further management.

### 2.4 Sample size

Sample size considerations were made with respect to the precision of the prevalence estimate. According to the literature (39) and data from Austria (40), the prevalence of CKD in the at-risk population is between 14% and 24%. Since patients were recruited from different GPs practices, the precision of the prevalence estimate was influenced by the design effect DE, whereby DE = 1 + (m - 1)*ICC and m is the average cluster size and ICC the intraclass correlation coefficient. Assuming a comparable prevalence in Styria, with a sample size of n = 1,000, an ICC of 0.01 and an average cluster size of m = 30 to m = 100 a prevalence of 15% to 35% could be estimated with a 95% confidence interval of ± 2.5 to ± 4.2.

### 2.5 Statistical methods

Continuous baseline variables were reported as median and interquartile range or mean and standard deviation and categorical variables were reported as absolute and relative numbers. The prevalence of previously undetected CKD in the population at-risk and the 95% confidence interval were estimated using a mixed-effects logistic regression model (random effect: GP practice) based on the available sample. To evaluate the effect of the primary care practices, a sensitivity analysis was performed, excluding the random effect primary care practices, by calculating the relative proportion of affected individuals and the corresponding 95% confidence intervals.

In addition to the primary outcome, the influence of baseline characteristics on the presence of CKD was analyzed using logistic regression (outcome: CKD yes/no). Primary care practice was included in the models as a random effect. Variables with a p < 0.2 in univariable analyses were included in multivariable analyses. Before entering in the final model these variables were tested for multicollinearity. The resulting potential predictors for CKD were entered in the final analysis. Backward selection was used to determine the final model with independent significant predictors. All data management and analyses were performed using SAS (version 9.4) and R (version 4.2.1).

## 3 Results

**FIGURE 2:**
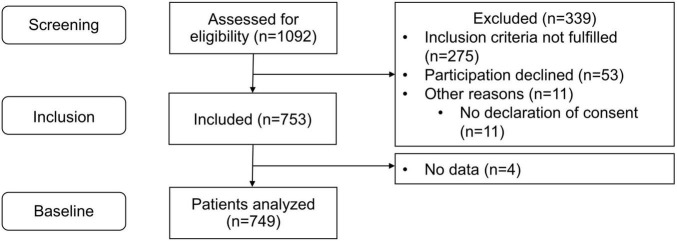
Patient flow chart.

Between January 2021 and December 2022, 1,092 patients in 33 primary care practices were screened for eligibility (5 to 97 per practice; median: 28). Of these, 339 could not be included (did not fulfill inclusion criteria: *n* = 275, no informed consent: *n* = 53, other reasons: *n* = 11) and no data were collected for a further four patients. Overall, 749 patients (3 to 65 patients per practice, median: 23) were included in the analysis (Figure 2).

Mean age of analyzed patients was 56.2 ± 6.4 years, 46.1% (*n* = 345) were female, and the most common risk factors were arterial hypertension (77.3%) and obesity (52.5%). Antihypertensives were the most frequently taken medications (73.9%) (Table 2).

**TABLE 2:** Baseline characteristics.

		*n* (%)
Age		56.2 ± 6.4
Sex	Male	404 (53.9%)
	Female	345 (46.1%)
Arterial hypertension	No	167 (22.7%)
	Yes	569 (77.3%)
Obesity	No	343 (47.5%)
	Yes	379 (52.5%)
Diabetes mellitus (Type 1 or Type 2)	No	409 (55.3%)
	Yes	331 (44.7%)
History of cardiovascular disease	No	629 (85.1%)
	Yes	110 (14.9%)
End-stage kidney disease in the family	No	690 (95.4%)
	Yes	33 (4.6%)
Antihypertensive drugs	No	195 (26.1%)
	Yes	551 (73.9%)
Other long-term medications^a^	No	257 (34.8%)
	Yes	482 (65.2%)
Statins	No	441 (59.2%)
	Yes	304 (40.8%)
Antidiabetic drugs	No	460 (62.1%)
	Yes	281 (37.9%)
Non-steroidal anti-rheumatic drugs (NSAR)	No	648 (88.4%)
	Yes	85 (11.6%)

^a^“Other long-term medications”: Platelet aggregation inhibitors (TAH), anticoagulants, proton pump inhibitors (PPI), psychotropic drugs, uricostatic drugs (allopurinol), GABA analogue, morphine/opiate, vitamins/trace elements (calcium, iron, vitamin D, folic acid), thyroid medication, bronchodilators, analgesics.

Overall, 150 at-risk patients fulfilled the criteria for CKD. Mean age of these patients was 57.6 ± 5.9 years, 41.3% (*n* = 62) were female, and the most common risk factors were arterial hypertension (82.9%) and obesity (59.7%). Antihypertensives were the most frequently taken medications (82.7%) (Supplementary Table 2). An ACR ≥ 30 mg/g (A2-A3) was detected in 121 patients and an eGFR < 60 ml/min/1.73 m^2^ ( ≤ G3) in 39. EGFR was < 30 ml/min/1.73 m^2^ in three patients and between 30 and 59 ml/min/1.73 m^2^ (stage G3) in 36. Ten patients were diagnosed with both albuminuria (A2–A3) and reduced kidney function ( ≤ G3). The prevalence of previously undetected CKD in an at-risk population was estimated to be 20.1% (95% CI: 17.1–23.6). The estimated prevalence was highest in patients with diabetes mellitus (25.6%, 95% CI: 20.0–32.0), followed by patients with obesity (22.4%, 95%CI: 17.0–28.9), hypertension (21.3%, 95% CI: 18.1–24.8) and cardiovascular diseases (20.0%, 95% CI: 13.6–28.5).

Univariable predictors of CKD in an at-risk population were higher age (Odds Ratio (OR): 1.05, 95%CI: 1.02–1.08, *p* = 0.003), diabetes mellitus (OR 1.83, 95%CI: 1.27–2.64, *p* = 0.001), antihypertensives (OR: 1.89, 95%CI 1.19–2.99, *p* = 0.007), statins (OR: 1.49, 95%CI 1.04–2.14, *p* = 0.029), antidiabetics (OR: 1.87 95% CI 1.30–2.68, *p* = 0.001) and other long-term medications (OR: 1.65, 95% CI: 1.11–2.47, 0.014) (Table 3). Other variables with *p* < 0.2 were sex (higher risk for men), arterial hypertension and obesity. Higher age (OR 1.06, 95%CI: 1.02–1.09), diabetes mellitus (OR 1.65, 95% CI: 1.12–2.30) and obesity (OR: 1.55, 95% CI: 1.04−2.30) turned out to be independent significant predictors of previously undetected CKD in the multivariable analysis of the at-risk population.

**TABLE 3:** Analysis of potential predictors of CKD in an at-risk population (uni- and multivariable results).

	Univariable		Multivariable	
	*P*-value	OR (95%CI)	Sig.	OR (95%CI)
Age (years)	0.003	1.05 (1.02–1.08)	0.001	1.06 (1.02-1.09)
Sex (reference (ref.): male)	0.194	0.79 (0.55–1.13)		
Arterial hypertension (ref.: no)	0.074	1.53 (0.96–2.46)		
Obesity (BMI > 30 kg/m^2^) (ref.: no)	0.059	1.44 (0.99–2.09)	0.033	1.55 (1.04-2.30)
Diabetes mellitus (Type 1 or Type 2) (ref.: no)	0.001	1.83 (1.27–2.64)	0.012	1.65 (1.12-2.43)
History of cardiovascular disease (reference: no)	0.945	1.02 (0.61–1.69)		
End-stage kidney disease in the family (ref.: no)	0.497	1.33 (0.59–3.01)		
Antihypertensive drugs	0.007	1.89 (1.19–2.99)		
Other long-term medications	0.014	1.65 (1.11–2.47)		
Statins	0.029	1.49 (1.04–2.14)		
Antidiabetic drugs	0.001	1.87 (1.30–2.68)		
Non-steroidal anti-rheumatic drugs (NSAR)	0.738	0.91 (0.51–1.61)		

## 4 Discussion

### 4.1 Summary

The nephrology awareness study “kidney.care 2.0” included 749 Austrian patients between 40 and 65 years of age and with one or more risk factors (hypertension, diabetes, cardiovascular disease, obesity or family history of end-stage kidney disease). The prevalence of previously undetected CKD was estimated at 20.1%. In a multivariate analysis, age, diabetes and obesity were independent predictors of CKD. Albuminuria (A2-A3) was present in the majority of patients with previously undetected CKD. Most patients with previously undetected eGFR < 60 ml/min/1.73 m^2^ were in stage G3.

### 4.2 Comparison with existing literature

Our results confirm those of previous research that the prevalence of CKD in patients with additional risk factors such as arterial hypertension, diabetes, obesity, etc. (2, 5–8, 10, 13, 41–47) is high. However, in our multivariate analysis, only age, diabetes and obesity remained significant predictors. Although gender and arterial hypertension were not predictive of CKD in our study of over 700 patients, the odds ratios tended in the same direction as previous prevalence studies involving high number of patients that reported that female sex (6, 43, 44, 48, 49) and arterial hypertension (5, 6, 10, 41, 43, 47, 50) were both predictive of CKD, e.g. the systematic analysis for the Global Burden of Disease Study (49) or the National Health and Nutrition and the Examination Survey (47). However, in the systematic review by Mills (51), there was no difference in CKD prevalence between females and males in younger age groups.

It is also unsurprising that age is an independent predictor of CKD (5, 6, 43, 52, 53), as was also seen in our study population of middle-aged patients. However, it should be taken into account that a decline of 6–7 ml/min/1.73 m^2^ per decade from of the age of 35 to 40 years is part of normal ageing (54). It is therefore understandable that some researchers recommend adjusting the 60 ml/min/1.73 m^2^ threshold according to age (55, 56). For example, in a UK study in which Shardlow et al. followed up on 1,741 patients with mild (stage G3) CKD in 32 GP practices, the majority had stable kidney function after 1 and 5 years, and only a very small minority developed end-stage kidney disease, with 18% showing a less severe progression after 5 years (57). The authors concluded that the intervention should focus on slowing the progression of CKD and reducing the number of cardiovascular events in a small group of patients at high risk of adverse outcomes. They therefore recommended an age-adjusted definition of CKD to avoid considering a large group of people with age-related decline in GFR as ill.

In our study population, obesity was an independent predictor of CKD, which is in agreement with other studies (8, 50). However, it remains unclear whether obesity in CKD patients is also associated with future cardiovascular diseases. One systematic review and meta-analysis of observational cohort studies and randomized controlled trials that included over 27,000 individuals without end-stage CKD provided evidence that obesity was not significantly associated with cardiovascular events (2).

Diabetes is a major risk factor in the development of CKD (5, 6, 10, 41–43, 46, 48) and is the leading cause of end-stage kidney disease (58). A recent study by Ohkuma showed that both decreases in eGFR and increases in ACR over 2 years, were significantly associated with a higher risk of myocardial infarction, stroke, cardiovascular death, major kidney events and all-cause mortality in patients with type 2 diabetes. The study results suggest that a combined assessment of clinically meaningful changes in both eGFR and ACR improves the risk stratification of people with type 2 diabetes with regard to their risk of experiencing major cardiovascular and kidney events (59).

In the “kidney.care 2.0” study, albuminuria was present in the majority of our patients with previously undetected CKD, which agrees with the results of previous screening studies in high-risk individuals (40–42). It is well known that predictive models for end-stage kidney diseases are significantly limited by a lack of external validity and efficacy (60). Nonetheless, the classification into different stages provides helpful guidance and supports communication with patients. In 2019, a new predictive model was adapted from the Kidney Failure Risk Equation (KFRE) (38) and published for the primary care setting (61). It is based on a British cohort and assesses more accurately the risk of end-stage kidney disease in primary care after 2 and 5 years, thus reducing the number of unnecessary referrals and increasing the number of earlier referrals in those at high risk of developing end-stage kidney disease (61). Even though most stage G3 CKD patients never progress to end-stage kidney disease, they are more likely to experience other adverse events such as those linked to cardiovascular diseases (1–3). Based on an adapted KFRE model that took into account cardiovascular comorbidities, the 5-year risk of progression to kidney failure in our patient group was stratified as high (62).

### 4.3 Screening and treatment strategies

Symptoms of CKD are often lacking and awareness of the disease is generally low, not only in primary care (12, 14, 18), but also in the hospital setting (14, 17, 63, 64). We therefore chose a pragmatic approach to screening in Austrian GP practices, which is the place where most asymptomatic patients with mild CKD are first identified. Unlike other countries, no database of electronic health records exists in the primary care setting in Austria (13, 62, 65). In our study, patients we identified as at high risk were screened and their health care managed according to our adapted “kidney.care 2.0” program. The written materials provided in the training course recommend doctors ensure their patients undergo regular laboratory tests. They also recommend optimizing treatment by seeking close support from nephrologists via a telephone hotline, and through preferential access to the outpatient clinic responsible for monitoring progression. Similar procedures were also performed in other studies (20, 28) in which only high-risk patients were screened for CKD in a primary care setting because screening those with no risk factors was considered to be excessive (66). Furthermore, it is not only GPs that are unaware of the dangers of kidney disease but also the patients themselves. In a survey in the UK, it was shown that only one in two people knew that kidneys produce urine and only 12% of the population knew that kidneys play a role in processing medicines (67). Furthermore, Weckmann et al. (15) described in a German population cohort study that only 9% of participants with reduced GFR reported having CKD. Against this background, disclosure of kidney dysfunction to patients appears advisable in that it would probably encourage them to adhere to appropriate kidney protective therapies and life style modifications (exercise and diet), and raise their awareness of the need to both adjust doses of kidney excreted drugs and avoid nephrotoxic substances. Considering that a systematic review that included several educational interventions for patients with CKD has shown that, although inconsistent, different educational interventions lead to some improvement in patient reported and relevant outcomes, there would also appear to be a need for more patient education and information (68).

Although there is no robust evidence for the usefulness of screening and monitoring strategies for CKD (32, 69, 70), several guidelines (24, 71) and consensus statements (28, 29, 72) on screening and monitoring nonetheless exist. However, it should be borne in mind that over-testing can lead to harm through the incorrect labeling and reclassification of patients, as this may be associated with unnecessary changes in medications and possible additional costs (25). These uncertainties highlight the importance of shared decision-making between doctors and patients.

We implemented the “kidney.care 2.0” program because experience in various countries has shown that in primary care settings, full CKD screening and diagnosis/detection (i.e. assessment of both eGFR and ACR) is rarely routinely performed for at-risk patients (14, 18, 73). Several factors may help explain this, including a lack of awareness of the importance of early diagnosis of CKD among GPs (16). For this reason, the “kidney.care 2.0” program also aimed to increase awareness through educational interventions. In Styria, Austria, a further reason may be that the health care system does not foresee reimbursement of the cost of ACR testing. Similarly, resource constraints may mean that laboratories do not automatically, and GPs do not routinely calculate eGFR.

Currently, patients with CKD and/or albuminuria are generally prescribed ACE-Inhibitors or Angiotensin Receptor blockers (37). Studies like the RENAAL- study by Brenner showed that such standard care leads to a reduction in eGFR loss and slows the progression to end-stage kidney disease in patients with type 2 diabetes (67). In recent years, the armamentarium used in the treatment of CKD with or without albuminuria has been expanded through the use of the new sodium glucose transport 2 (SGLT2)-inhibitor. Several studies have shown that it dramatically slows the deterioration of kidney function in diabetic and non-diabetic patients, as well as having a very positive impact in patients with chronic heart failure (74). It is therefore of the utmost importance that CKD and/or albuminuria are identified at an early stage, as nephrologists now have a very effective means of treating CKD (75). Other drugs for the treatment of diabetic albuminuric kidney disease are in the pipeline (76), which are expected to further slow the progression of CKD, and lead to a dramatic reduction in cardiovascular events (67, 77).

The first part of the “kidney.care 2.0” study aimed to identify CKD patients at increased risk of primarily adverse cardiovascular outcomes and to support them with subsequent monitoring and targeted clinical management. It remains to be seen whether we will see a change in prescribing behavior and slower progression to CKD in this patient population (33) in the 12-month follow-up period.

### 4.4 Strengths and limitations

The “kidney.care” project was launched in 2016 (30) and has implemented several initiatives aimed at increasing awareness of the importance of screening in the primary care setting. The “kidney.care 2.0” program was launched in 2021 and has attempted to increase awareness through updated training courses, further education via public media, and on-site visits to GPs. In a previous publication, various evaluation methods for the awareness program “kidney.care” were discussed and we came to the conclusion that it should be embedded in a disease management program, which does not yet exist in Austria (32). In addition, unlike the UK (78), the USA (79) and Canada (20), no large-scale national societies and initiatives exist.

Even though risk factors such as hypertension, a history of cardiovascular diseases and a family history of end-stage kidney disease have been shown to be predictors in several prevalence studies (2, 5, 6, 10, 41, 43, 47, 50, 80, 81), we could not provide similar evidence in our study population (33), which may be due to its small size.

As part of “kidney.care 2.0”, only one laboratory test was carried out to determine CKD on the basis of a reduced eGFR and an elevated albumin-creatinine ratio (ACR), with a further measurement of ACR not being undertaken until three months later in case of first-time albuminuria. It was therefore impossible to draw a conclusion on the permanence of impaired kidney function. Furthermore, overdiagnosis cannot be ruled out as it is unclear to what extent the lack of repeated measurements of eGFR may have affected the result. Another reason for a potential overestimate might be the biological and analytical variability of eGFR and ACR (71). Therefore, the planned follow-up survey will determine whether the CKD cases detected at baseline can be reconfirmed. This analysis will help to distinguish persistent CKD cases from those influenced by transient variations in kidney function and albuminuria.

A further limitation of the present study is that eGFR was calculated using the 2009 Epidemiology Collaboration (CKD-EPI) equation (36) rather than the updated CKD-EPI equation published in 2021 (82). This discrepancy may affect the comparability of our study results with other publications.

It should be noted that one of this study’s limitations is that when the “kidney.care” program was developed in 2016, we restricted patient inclusion to predefined risk groups in accordance with NICE Guidelines (83). We later decided to further restrict the program to include only middle-aged patients because of such practical considerations as the feasibility of the program for GPs and the availability of funding. In view of the continued implementation of the “kidney.care” program, it is imperative to assess the necessity to adapt the risk groups to be screened for CKD in accordance with the current KDIGO Guideline 2024 (71). Risk groups would then include, for example, patients with previous acute kidney injury, chronic inflammation and younger diabetic patients.

In our referral recommendations, we suggest implementing specific monitoring intervals, especially for those in whom CKD has not yet been detected. Our recommendations are based on consensus papers (84, 85) and the opinion of nephrology experts. However, as no high-quality studies have addressed the optimal frequency for testing patients with (or at high risk of developing) CKD, harm from over-testing cannot be ruled out (25, 69). While this may be a challenge for screening programs outside a trial setting, regular monitoring has clear advantages and is essential in a study context, particularly for the reliable detection of changes in renal function and the onset of CKD. Papers by Major (61) and Mosa (62) had not been published when our referral recommendations were adapted from the KFRE (38). It is possible that our recommendations will be adjusted to take account of the findings reported in these publications.

The COVID pandemic often made GPs unwilling to participate, which slowed down the recruitment of patients considerably. A further limitation of our study is that the sample of participating GPs was not representative.

Despite several extensions to the recruitment period and local support measures on-site, it became apparent in spring 2022 that the target of including 1,000 patients would not be reached. For this reason, the sample size calculation was carried out again in April 2022. The calculation revealed that a reduction to 700 patients would decrease precision from ± 2.5 to ± 4.2 to ± 3.1 to ± 5.1. This was considered acceptable.

In order to account for the effects on the results of differences between individual practices, a random effect was included in the statistical analysis. However, no systematic analysis of the influence of specific participating practices on the results was conducted. Furthermore, no systematic investigation into the socioeconomic characteristics of study participants and their influence on the results was performed. These factors could be subjects for further investigation in future studies. It is also important to note that the limited scope of the study meant that the study population was not as diverse as would have been desirable in terms of ethnicity.

### 4.5 Implications for research and/or practice

The aim of our study was to integrate pragmatic CKD screening into the routine workflows of GP practices and to raise awareness among GPs of the need for regular follow-up care of CKD patients. As this study was carried out in only one federal state of Austria, the next step is to implement it on a national scale, as was recommended when the Austrian periodic health examination was revised in 2019 (86). The European Society of Cardiology (ESC) Guideline 2021 on Cardiovascular Disease Prevention in Clinical Practice 2021 also proposes a cardiovascular risk assessment in at-risk individuals and even considers a systematic or opportunistic cardiovascular risk assessment in men aged > 40 years and postmenopausal women or women > 50 years (87). The Council of the European Renal Association recommends that a cardiovascular risk assessment in the general population should include the assessment of eGFR und ACR (88).

In addition, with the support of the Austrian Society of Nephrology (89) and the Austrian Society of General Practice and Family Medicine (90), we will further sensitize GPs to the necessity of keeping a close eye on people at increased risk of CKD, as most patients with CKD can be managed in a primary care setting, as long as the option of referrals to nephrologists or of contacting nephrologists is also available. The “kidney.care 2.0” program is a perfect basis on which to expand existing cooperation.

Future research should investigate the efficacy of CKD screening program on patient-relevant clinical outcomes. This is why the U.S. Preventive Services task force (USPSTF) is planning a systematic review of the effects of screening for CKD in order to update its recommendations. A key question is the effect of screening for CKD versus no screening on clinical outcomes in asymptomatic adults without known CKD (91).

## 5 Conclusion

In this milestone Austrian study, pragmatic, risk-based, targeted screening for CKD in primary care was able to identify a large number of patients with previously undetected CKD. Further research should be conducted to find out whether risk-based, targeted screening and subsequent monitoring for CKD in primary care has the potential to optimize the care of middle-aged patients, slow the progression to kidney failure, and prevent cardiovascular events.

## Data Availability

The original contributions presented in the study are included in the article/Supplementary material, further inquiries can be directed to the corresponding author.
