# Supplementary material for: Prevalence of undetected chronic kidney disease in high-risk middle-aged patients in primary care: a cross-sectional study
Source: Front Med (Lausanne). 2024 Aug 13;11:1412689. doi: 10.3389/fmed.2024.1412689 (PMC11347449; doi:10.3389/fmed.2024.1412689)
Supplement: Supplementary file 1 [file Table_1.DOCX]

Supplementary Material

# Supplementary Tables

**Supplementary Table 1.** Detailed description of recruitment and screening

| General practitioner recruitment | In order to obtain the requisite number of 30 to 40 collaborating GPs, a variety of recruitment measures were implemented between January 2021 and November 2022.  Recruitment was conducted by sending by out a written invitation by e-mail or post to all Styrian GPs under contract to the biggest Statutory Austrian Health Insurance Fund (Österreichische Gesundheitskasse), to all GPs that are members of the Styrian Society of General Practice and Family Medicine, and to about 110 GPs listed in the research network of our Institute of General Practice and Evidence-based Health Services Research.  In addition, our study was presented at regional medical training events and congresses for general practitioners (GPs).  Information about the content of the study was further disseminated via relevant Austrian healthcare journals. |
| --- | --- |
| Screening and patient recruitment process | To avoid selection bias, we aimed to consecutively screen all at-risk patients aged 45 to 65 years that attended a particular practice during an eight-week period until 24 to 40 eligible patients had been identified. GPs could choose when the eight-week period best suited them as along as it was between January 1, 2021 and December 31, 2022. Due to the Covid-19 pandemic, it was possible for GPs to extend the eight-week period in agreement with the study team. |
| Screening list | Each GP received a screening list on which to record details of all screened patients, including the date of screening, patient screening number, first and last names, date of birth, and the final decision on their inclusion or exclusion from the study. To ensure data privacy, the screening list remained in the participating GP practices. |
| Screening questionnaire and written informed consent | To check patient’s eligibility, a screening questionnaire was completed by the GP. This included the date of screening, GPs’ study ID, the patient’s screening number, age, sex, and the study's inclusion and exclusion criteria. Eligible patients were invited to participate in the study “kidney.care 2.0” during their doctor-patient consultations. If a patient agreed to participate in the study, written informed consent was obtained. |

**Supplementary Table 2.** Characteristics of patients with CKD compared to patients without CKD

|  |  | Total | *CKD* | |  |
| --- | --- | --- | --- | --- | --- |
| Patient characteristics (number of patients with available data) |  |  | *No* | *Yes* | *p-value* |
| Sex (n=749) | male | 404 (53,94%) | 316 (52,8%) | 88 (58,7%) | .203 |
|  | female | 345 (46,06%) | 283 (47,2%) | 62 (41,3%) |  |
| Age Groups (n=749) | 40 - <50 | 138 (18,42%) | 119 (19,9%) | 19 (12,7%) | .044 |
|  | 50 - <60 | 357 (47,66%) | 287 (47,9%) | 70 (46,7%) |  |
|  | 60 - <70 | 254 (33,91%) | 193 (32,2%) | 61 (40,7%) |  |
| Diabetes mellitus  (Type 1 or Type 2) (n=740) | No | 409 (55,27%) | 345 (58,3%) | 64 (43,2%) | .001 |
|  | Yes | 331 (44,73%) | 247 (41,7%) | 84 (56,8%) |  |
| Arterial hypertension (at least 3 months >140/90mmHg) (n=736) | No | 167 (22,69%) | 142 (24,1%) | 25 (17,1%) | .065 |
|  | Yes | 569 (77,31%) | 448 (75,9%) | 121 (82,9%) |  |
| Obesity (BMI >30 kg/m²) (n=722) | No | 343 (47,51%) | 287 (49,2%) | 56 (40,3%) | .082 |
|  | Yes | 379 (52,49%) | 296 (50,8%) | 83 (59,7%) |  |
| History of cardiovascular disease  (n=739) | No | 629 (85,12%) | 505 (85,2%) | 124 (84,9%) | .926 |
|  | Yes | 110 (14,88%) | 88 (14,8%) | 22 (15,1%) |  |
| End-stage kidney disease in the familiy (n=723) | No | 690 (95,44%) | 556 (95.7%) | 134 (94.4%) | .450 |
|  | Yes | 33 (4,56%) | 25 (4.3%) | 8 (5.6%) |  |
| Antihypertensive drugs (n=746) | No | 195 (26,14%) | 169 (28,4%) | 26 (17,3%) | .005 |
|  | Yes | 551 (73,86%) | 427 (71,6%) | 124 (82,7%) |  |
| Statins (n=745) | No | 441 (59,19%) | 364 (61,2%) | 77 (51,3%) | .032 |
|  | Yes | 304 (40,81%) | 231 (38,8%) | 73 (48,7%) |  |
| Non-steroidal anti-rheumatic drugs (NSAIDs) (n=733) | No | 648 (88,40%) | 516 (88,2%) | 132 (88,4%) | .694 |
|  | Yes | 85 (11,60%) | 69 (11,8%) | 16 (10,8%) |  |
| Antidiabetic drugs (n=741) | No | 460 (62,08%) | 385 (65,1%) | 75 (50,0%) | <.001 |
|  | Yes | 281 (37,92%) | 206 (34,9%) | 75 (50,0%) |  |
| Other long-term medication (n=739) | No | 257 (34,78%) | 218 (36,9%) | 39 (26,2%) | .011 |
|  | Yes | 482 (65,22%) | 372 (63,1%) | 110 (73,8%) |  |
